# The Characteristics of People Requesting HIV Antibody Tests at Public Health Centers in Japan

**DOI:** 10.2188/jea.14.10

**Published:** 2005-03-18

**Authors:** Teruki Watanabe, Yosikazu Nakamura, Toshihide Kidokoro, Emi Shimazaki, Yoshiharu Hasegawa, Yoshitaka Tamura, Shinichi Tanihara, Shuji Hashimoto

**Affiliations:** 1 Tochigi Prefectural Institute of Public Health and Environmental Science; 2 Department of Public Health, Jichi Medical School; 3 Division of Health and Prevention, Nakano City Public Health Center; 4 Infectious Disease Control Section, Medical Service Division, Bureau of Public Health, Tokyo Metropolitan Government; 5 Sagamihara Public Health Center; 6 Infectious and Incurable Diseases Administration Division, Department of Public Health and Welfare, Osaka Prefectural Government; 7 Department of Environmental Medicine, Shimane Medical University; 8 Department of Hygiene, Fujita Health University School of Medicine

**Keywords:** HIV, HIV antibodies, acquired immunodeficiency syndrome, risk-taking, public health centers

## Abstract

BACKGROUND: The epidemiologic features of those who requested to undergo the human immunodeficiency virus (HIV) antibody test at public health centers in Japan are still ambiguous, although as a group, they are probably at a high risk to be infected.

METHODS: Between April 2001 and March 2002, 14,900 persons visited 131 public health centers that cooperated with this study in relation to the HIV antibody tests. A questionnaire was given to 8,972 persons who agreed to participate in the survey and 5,079 (56.6%) returned the form. Excluding those filled out by persons whose true intent in undergoing the test was the diagnosis of hepatitis C, 4,102 questionnaires were analyzed, individual characteristics examined, and first time visitors and repeaters were compared to assess their behavior and the reasons for of undergoing the test.

RESULTS: There were 2,515 (61.3%) males and 1,587 (38.7%) females. The largest age group was composed of 25 to 29 year-olds. Repeaters accounted for 27.2% of all the males and 21.3% of all the females. Their main reason for undergoing the test was anxiety about having contracted an HIV infection through sexual contact with a person of the opposite sex. The proportion of those having sexual contact with males was significantly higher among male repeaters (14.1%) than first timers (8.0%). Among females, the proportion of those who had experience of sexual contact with many and unspecified males was significantly higher for repeaters (39.6%) than first timers (27.3%).

CONCLUSION: It was evident from this study that repeaters exist among those who seek to be examined for possible contraction of HIV: they are characterized by risk-taking behavior in contracting an HIV infection through sexual contact.

In Japan, human immunodeficiency virus (HIV) antibody testing programs have been conducted at all public health centers since 1987. The social principle to help patients with HIV infections or AIDS was established in Japan when the first Japanese AIDS patient gave appropriate authorization (1986) or surveillance for these diseases was started by law (1989). Since then, the number of tests conducted was 48,754 and 69,924 in 2000 and in 2001, respectively.^1^ In the past, the trend in the number of persons requesting to be tested was investigated in only limited areas, such as some prefectural zones,^2^^,^^3^ because of a general rule concerning a patient’s privacy. Thus no research has been conducted that would reveal the characteristics (e.g., sex, age, and reasons for wanting to undergo the test) of persons requesting an HIV serodiagnosis.

Moreover, the number of those who sought a serodiagnosis was not strictly in accordance with the general trend in the incidence of HIV infectious or acquired immunodeficiency syndrome (AIDS) because of the principle that no person who wishes to undergo the test will be denied even when he is only anxious about contracting an HIV infection but has no definitive symptoms.^2^ In conducting the HIV antibody testing program, it is important not only to diagnose the HIV infection: it is also to reduce anxiety or to alter one’s behavior to prevent future infections.^4^ Thus, to conduct more effective programs, it is important to understand individual backgrounds and characteristics.

At the scene of the actual test, the person in charge of testing may recognize those who have been tested earlier. Although the difference in the characteristics between the first timers and repeaters is deemed important, no organized studies have been conducted. Therefore the thrust of the current study was to delineate the characteristics of persons who sought to undergo the test, determine the number of the so-called repeaters, and outline the behavior patterns that may result in contracting an HIV infection.

## METHODS

The subjects were those persons who sought to undergo the HIV antibody test at public health centers, which cooperated with this study for a one-year period between April 2001 and March 2002. Before the survey, we had asked each center to participate in this study and obtained cooperation from 131 public health centers in 33 prefectures, which were scattered throughout Tohoku, Kanto, Chubu, Kinki, Chugoku, Shikoku, and Kyushu (except Hokkaido). At that time, there were 592 public health centers in all 47 prefectures and the participating centers were 22.1% of the total (131/592).

A questionnaire, in the form of an unsigned response, was adopted. However, the codes for the respective prefectures and public health centers had been entered on the form beforehand so that the response rates could be calculated later. Handed out by the staff at each center, the prospective respondent was instructed to mail it directly to the research office.

During the study period, 14,900 persons requested the HIV antibody test at the participating public health centers; and the staff at the center requested cooperation with this study by filling out the form (those whose true purpose for undergoing the test was judged to have the hepatitis C virus antibody test were excluded). A full explanation—including the fact that the respondent could not be traced nor could the contents of the response be examined because the form was unsigned and mailed directly to the research secretariat (Department of Public Health, Jichi Medical School)—was given to each prospective respondent. The questionnaire and a self-addressed stamped return envelope were given to 8,972 persons (60.2% of all those seeking to take the test) who had expressed an intention to cooperate.

Through the questionnaire, information on demographic data, reason for undergoing the test, and behavior, including that possibly culminating in getting an HIV infection, age, sex, nationality (Japanese or foreign national), the date of blood specimen collection, the reply date, and the name of the public health center that the respondent visited, was acquired.

The number of visits made (including the one for the current HIV antibody test) was asked. A person who had responded that the current visit was “for the first time” was called “a first timer,” and one who listed a number of tests was classified as “a repeater.”

The true reason for requesting the diagnostic test at this time was sought by posing questions on “anxiety about an HIV infection because of sexual contact with a person of the opposite sex,” “anxiety about an HIV infection because of sexual contact with a person of the same sex,” “necessity to present a negative result on an HIV antibody test (to a sexual partner, a company, or others),” “anxiety about an HIV infection because of a medical procedure that has been conducted (such as a blood transfusion),” and “anxiety about having contracted an HIV infection via an illicit drug injection.” To a repeater, questions on the last test were asked by classifying the time by “less than one month,” “less than six months,” “less than one year” and “one or more years previously.” The identification of the institution where the previous test was conducted was made by posing questions, such as the previous test was conducted “at the same public health center,” “another public health center,” “a medical institution dedicated to conducting an HIV antibody test program,” “a medical institution otherwise intended for another purpose,” “a medical institution designed to conducted medical checkups for pregnant women,” and “others.”

For risk-taking behavior over a period of one year before seeking the current test, the following were answered by “yes” or “no”: “ear- piercing for wearing earrings or tattooing”; “being accidentally stabbed with a used injection needle”; “having sexual contacts with many and unspecified persons of the opposite sex”; “having sexual contact with other men” (a question only for men); “having been informed of a positive result from an HIV antibody test”; “having injected drugs, such as narcotics or stimulants”; and “having had a sexual contact with the person who corresponded to any of the conditions given above.” These items were adopted from those related to HIV infections that are asked when blood is donated at the Japan Red Cross Society.

Unaware at the onset of the study, a hepatitis C virus antibody test program was conducted at public health centers throughout the country during May through October 2001. Because this HCV antibody test was given free of charge simultaneously with the HIV antibody test, it became necessary to take into consideration the possibility that the group ostensibly composed of those who wished to have the HIV antibody test unwittingly included individuals who wanted to be tested for the presence of the hepatitis C virus antibody. For this reason, the rule for selecting the subjects was modified so that the questionnaire would not be handed out to those who wished to have the HCV antibody test conducted. This modification was made at the 64 public health centers where the personnel had an opportunity to find out whether the would-be subject’s real intention was to be tested for hepatitis C.

The subjects’ characteristics, i.e., sex and age, were examined. The proportion of the repeaters was calculated by their sex, age, and the area where they were located and analyzed by chi-square tests. Also, the repeaters were stratified by gender and the timing and place of the last test were found out. Moreover, the proportion of risk-taking behavior was calculated and compared between the first timers and repeaters and the data were analyzed by using the chi-square tests. All statistical analyses were conducted by employing SPSS for Windows ver.11. The significance level was set at 5%.

## RESULTS

Table 1 shows the state of recovery of the questionnaires that were distributed. They were given to 8,972 of 14,900 persons who visited the public health center (60.2% of all those who requested the HIV test). Among these, the forms were returned by 5,079 persons for a recovery rate of 56.6%.

**Table 1.** The state of recovery of the questionnaires

Area	No. of requested HIV antibody tests ^a^ (*α*)	No. of individuals visiting public health centers (*β*)		No. of distributed questionnaires (*γ*)		No. of retrieved questionnaires (*δ*)	
Hokkaido	1,716	0	( 0.0%) ^b^	-	( - ) ^c^	-	( - ) ^d^
Tohoku	2,398	945	( 39.4%)	832	( 88.0%)	501	( 60.2%)
Kanto (except Tokyo)	16,385	2,874	( 17.5%)	2,210	( 76.9%)	1,470	( 66.5%)
Tokyo	9,884	3,010	( 30.4%)	1,696	( 56.3%)	852	( 50.2%)
Chubu	12,783	1,550	( 12.1%)	627	( 40.5%)	418	( 66.7%)
Kinki	15,552	3,733	( 24.3%)	1,653	( 44.3%)	962	( 58.2%)
Chugoku and Shikoku	3,735	704	( 18.8%)	536	( 76.1%)	306	( 57.1%)
Kyushu	7,091	2,084	( 29.4%)	1,418	( 68.0%)	568	( 40.1%)

Total	69,544	14,900	( 21.4%)	8,972	( 60.2%)	5,079	( 56.6%)

a: Ministry of Health, Labor and Welfare, AIDS Surveillance Committee, AIDS Surveillance Committee Report, October 30,2002.

b: *β*/*α*

c: *γ*/*β*

d: *δ*/*γ*

Suitable for analysis were 4,102 persons: 968 (19.1% of all respondents) were excluded because their real intention in requesting the test was “anxiety about an HIV infection because of a medical procedure (such as a blood transfusion)” or choosing “others” with a clear mention of hepatitis C virus antibody test as the purpose of their visit. In addition, 9 others were excluded because no gender was specified on the questionnaire.

**Figure 1.**
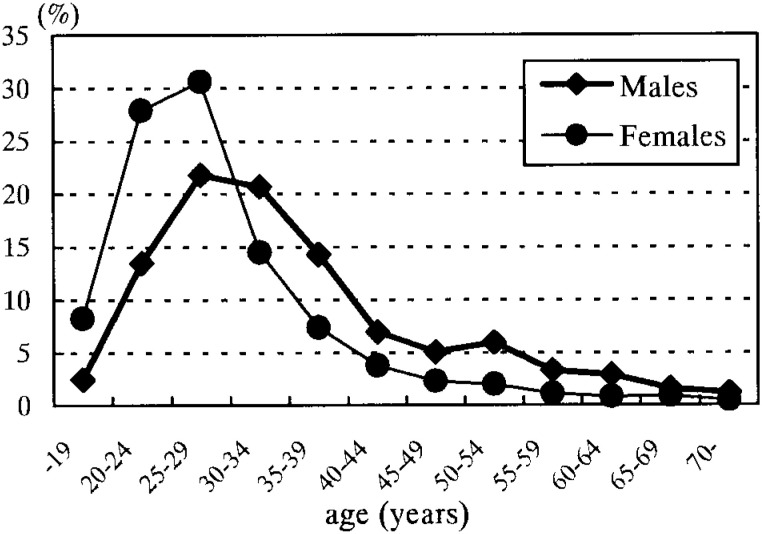
Proportional age distributions of the subjects by sex

Of all the participants, the numbers of males and females were 2,515 and 1,587 (61.3% and 38.7%), respectively. The proportion of Japanese nationals was 97.7% (2,457/2,515 individuals) among males and 98.1% (1,557/1,587) among females. Figure 1 shows the distribution of the subjects by age. The largest numbers fell between 25 and 29 years, which accounted for 548 and 485 (21.8 % and 30.6%), of all males and female, respectively. By observing the age and sex distribution, a pattern of dominance by a younger segment of the population and females over males was noted.

Table 2 shows the percentage of repeaters and sex distribution-27.2% (683/2,515) among males and 21.3% (338/1,587) among females-with the proportion of the males being significantly higher.

**Table 2.** Last time when repeaters were tested

Time	Males		Females	
Less than one month	34	( 5.0%)	11	( 3.3%)
Less than six months	119	(17.4%)	64	(18.9%)
Less than one year	74	(10.8%)	40	(11.8%)
One or more years previously	442	(64.7%)	220	(65.1%)
No answer	14	( 2.0%)	3	( 0.9%)

Total	683	( 100%)	338	( 100%)

Percentage of repeaters	27.2%	(683/2,515)	21.3%	(338/1,587)

**Figure 2.**
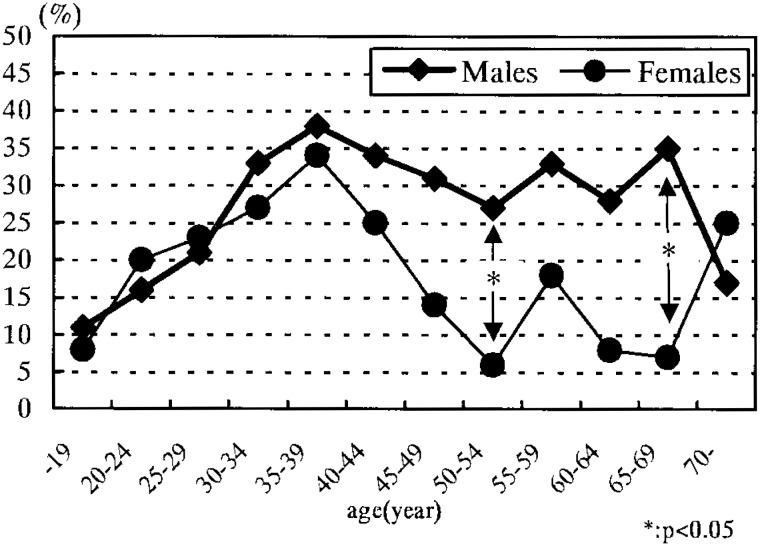
Proportion of repeaters among the subjects (stratified by age and sex)

Figure 2 shows that the proportion of repeaters was highest in the 35-39 age group for both sexes: 37.6% (135/359) among males and 33.9% (40/118) among females. In the age group under 40, the proportion of repeaters increased with age for both sexes. For the 50-54 and 65-69 year-olds, the proportion of repeaters among males was significantly higher.

Among the repeaters, the median number of times individuals sought to have the test was 2 and the maximum was 20. Table 2 shows that the most frequently given time by both sexes when the last test was run was “one or more years previously.” On the other hand, 22.3% of the repeaters returned within a short period of time for a test (within 6 months). Table 3 shows that for both sexes, the repeaters most frequently returned to the same public health center for the HIV antibody test and the proportion of these repeaters returning to the same public health center was significantly higher among males. The proportion of those who visited “medical institutions dedicated to conducting an HIV antibody test program” was higher among females.

**Table 3.** Last institution that repeaters visited

Institution	Males		Females	
At the same public health center	351	(51.4%)	128	(41.8%)

Another public health center	212	(31.0%)	93	(30.4%)

A medical institution dedicated to conducting an HIV antibody test program	74	(10.8%)	45	(14.7%)

A medical institution otherwise intended for another purpose	19	( 2.8%)	18	( 5.9%)

A medical institution designed to conducted medical checkups for pregnant women	-	( - )	32	( - )

Others	14	( 1.8%)	3	(4.6%)

No answer	15	( 2.2%)	8	(2.6%)

Total	683	(100%)	338	(100%)

Table 4 shows that for both sexes and for the first timers and repeaters, the most common reason given for the current test request was “anxiety about an HIV infection because of sexual contact with a person of the opposite sex.” Among males, the proportion of those who cited this or “necessity to present a negative result on an HIV antibody test” as the reason for requesting the test was greater for the first-timers than the repeaters. The proportion of those who cited “anxiety about an HIV infection because of sexual contact with a person of the same sex” was higher for the repeaters. Among females, the proportion of those who explained their need for the test by citing “anxiety about having contracted an HIV infection via an illicit drug injection” was higher among the first timers.

**Table 4.** Reason for the present testing

Reason	Males						Females					
	
	Total		First timers		Repeaters		Total		First timers		Repeaters	
Anxiety about an HIV infection because of sexual contact with a person of the opposite sex	1,918	(77.5%)	1,426	(79.6%)	492	(72.0%) **	1,156	(74.1%)	904	(74.0%)	252	(74.6%)

Necessity to present a negative result on an HIV antibody test	286	(11.6%)	221	(12.3%)	65	( 9.5%) *	174	(11.2%)	129	(10.6%)	45	(13.3%)

Anxiety about an HIV infection because of sexual contact with a person of the same sex	187	( 7.6%)	114	( 6.4%)	73	(10.7%) **	3	( 0.2%)	1	( 0.1%)	2	( 0.6%)

Anxiety about having contracted an HIV infection via an illicit drug injection	24	( 1.0%)	19	( 1.1%)	5	( 0.7%)	22	( 1.4%)	21	( 1.7%)	1	( 0.3%) *

Others	231	( 9.3%)	170	( 9.5%)	61	( 8.9%)	294	(18.8%)	249	(20.4%)	45	(13.3%)

Total	2,475	(100%)	1,792	(100%)	683	(100%)	1,560	(100%)	1,222	(100%)	338	(100%)

* : p<0.05, ** : p<0.01, between first timers and repeaters

Table 5 shows the proportion of experiences with risk-taking behavior. For each item, the reply was obtained from 78.7 to 88.5% of the males and 80.2 to 86.8% of the females. Regardless of sex or the frequency of seeking an HIV test, the most common experience was “having sexual contacts with many and unspecified persons of the opposite sex.”

**Table 5.** Percentage ^a^ of experiences with risky behavior that may lead to HIV infection

Reason ^b^	Males						Males					
	
	Total		First timers		Repeaters		Total		First timers		Repeaters	
	(n=2,475)		(n=1,792)		(n=683)		(n=1,560)		(n=1,222)		(n=338)	
Ear- piercing for wearing earrings or tattooing	63	( 3.2%)	51	( 3.5%)	12	( 2.2%)	181	(13.7%)	149	(14.4%)	32	(11.1%)

Being accidentally stabbed with a used injection needle	9	(0.5%)	6	(0.4%)	3	(0.6%)	29	( 2.3%)	24	( 2.4%)	5	( 1.8%)

Having sexual contacts with many and unspecified persons of the opposite sex	1,070	(48.6%)	768	(48.7%)	302	(48.5%)	407	(30.0%)	287	(27.3%)	120	(39.6%)**

Having sexual contact with other men	192	( 9.7%)	115	( 8.0%)	77	(14.1%)**	-	( - )	-	( - )	-	( - )

Having been informed of a positive result from an HIV antibody test	4	(0.2%)	2	(0.1%)	2	(0.4%)	3	(0.2%)	1	(0.1%)	2	(0.7%)

Having injected drugs, such as narcotics or stimulants	6	(0.3%)	4	(0.3%)	2	(0.4%)	6	(0.5%)	4	(0.4%)	2	(0.7%)

Having had a sexual contact with the person who corresponded to any of the conditions given above	557	(28.5%)	395	(27.9%)	162	(30.1%)	343	(27.3%)	263	(26.6%)	80	(29.7%)

a: The proportion was analyzed excluding those with no answer.

b: The items above are those asked at blood donation at the Japanese Red Cross Society Blood Center.

Multiple answers given about the experience stated for each item.

** : p<0.01, between first timers and repeaters of the same sex

When the repeaters and first timers were compared, the proportion of those having experiences of “having sexual contact with other men” was higher among the male repeaters. The proportion of those having experience with “having sexual contacts with many and unspecified persons of the opposite sex” was higher for the repeaters than first time females.

## DISCUSSION

This study is the first nation wide large-scale investigation of individuals seeking an HIV antibody test at a public health center between April 2001 and March 2002. Only 21.4% of 69,544 people who sought to have an HIV antibody tests^1^ visited public health centers that participated in this survey, which may be a limitation of this study. Because the subjects voluntarily participated , it cannot be considered a randomized study.

In examining the characteristics of the people who sought to have an HIV antibody test at the public health centers, it was noted first that male participants outnumbered females and the age group known for more accentuated sexual activity (those under 40 years) constituted the majority of this population segment. These features were similar to those reported in an earlier study that covered a limited area.^3^ The features noted in the current study were inclusion of a large number of women and young people and the existence of variations in the potential cause of the HIV infection, when compared to those people infected with HIV who were described in a survey of infectious diseases (recently conducted for one year).^5^ Because the questionnaire used in this investigation was written in Japanese, many of the respondents in this study were presumably Japanese nationals and one may assume that the characteristics presented here are those of Japanese who sought to be tested for an HIV infection at a public health center. It is known by experience that foreign nationals also visit public health centers to be tested for HIV infections and that research on these people has been conducted at specific health centers.^6^ The current study is inadequate in elucidating the health status of these foreign nationals in relation to this infection, which limits its scope as a nationwide report.

Second, one of the main thrusts of this study was to clarify the actual status of repeaters in the test program of this kind. Because of the anonymity of the participants, it has been impossible to ascertain even their existence until now. Thanks to this study, it became evident that 27.2% and 21.3% were repeaters among male and female respondents, respectively. Between the first timers and repeaters, their reasons for wanting to be tested for an HIV infection were different. The proportion of repeaters increased by age up to 40 years. The most frequently cited reason for wanting to have the test was anxiety about having an HIV infection that was transmitted through sexual contact. This was also quoted as the main reason for contracting an HIV infection among the persons who reported and were found to have an HIV infection or AIDS patients through a survey of infectious diseases.^5^ Moreover, among those who cited a “need for reporting seronegativity concerning the status of an HIV infection” as the reason for their current visit to a public health center, there were those who stated that the “negative result has to be reported so that they will be allowed to participate in wrestling and other combative tournaments.” Some of these people were being tested for the 20th time. In addition to the obvious need to be examined for medical reasons, the existence of such a necessity should also be kept in mind. It was possible that their anxiety about an HIV infection was not assuaged by past negative results because there were repeaters who had visited a testing facility that was different from the last institution that they visited or their behavior that caused the infection anxiety has been repeated. It was possible that repeaters hesitated to reply the questionnaire. So, bias might have arisen because of a difference in response rate between repeaters and first timers.

In analyzing the reason why an individual seeks to undergo an HIV test, the matter of the hepatitis C virus antibody test had arisen. To remove this influence, it was considered logical to exclude those who chose “anxiety about an HIV infection because of a medical procedure that has been conducted (such as a blood transfusion)” as a reason for their current desire to have the test performed on them. A possibility was pointed out: those who desired to know their serological status in relation to the hepatitis C virus antibody had been eliminated from the group desiring to undergo the HIV antibody test during the period when the two serological tests were conducted in a single program.^7^ In the current study, 64 public health centers did not hand out the questionnaire to those who were suspected to be the candidates for the hepatitis C virus antibody test. The forms were distributed only to 47.5% of those who visited these clinics (2,117/4,455) during the period when the hepatitis C virus antibody test was being conducted. The response rate was 64.2% (1,360/2,117) then. On the other hand, at the 60 public health centers that did not make a distinction between the two groups, the questionnaire was handed out to 62.1% (3,032/4,881) of those who sought to undergo the test during the same period, the response rate being 55.6% (1,686/3,032). Those who had intended to undergo serodiagnosis for hepatitis C are not likely to respond to the questions posed in the questionnaire: it is more likely that they were included in the group that failed to return the questionnaires. It was concluded that at the response stage, the influence of those who sought to undergo hepatitis C serodiagnosis was practically eliminated.

Finally, it was important to have a practical understanding of the risk-taking behavior of those seeking an HIV serodiagnosis because this behavior should be taken into consideration in implementing effective test programs, the establishment of which is one of the main purposes of this study. The proportion of those who had experience in taking the risk of contracting a sexually transmitted disease was comparatively high in repeaters of both sexes. It was thought important that intervention be made to correct their behavior to prevent future infections. In selecting questions to be posed, more general expressions were selected and the risk-taking behavior was not subdivided into more specific questions. The questionnaires may be blamed for a lack of specificity and inexactitude in evaluating the responses. For example, other studies on specific groups subdivided and estimated the risk-taking behavior by adding questions—such as one’s history of sexually transmitted diseases, the customary use of condoms before sexual contact, and the specificity of sexual partners.^8^ It should be added that although small in number, those who had been informed of their seropositive AIDS (HIV infections) status were found, regardless of their gender or repeat status in the current study. The finding constitutes important information in planning test programs such as this to improve the precision of the test and prevent accidents (such as needle punctures).
